# Proteome and microbiota analysis highlight *Lactobacillus plantarum* TWK10 supplementation improves energy metabolism and exercise performance in mice

**DOI:** 10.1002/fsn3.1635

**Published:** 2020-06-03

**Authors:** Yi‐Ming Chen, Chen‐Chung Liao, Yen‐Chun Huang, Ming‐Yi Chen, Chi‐Chang Huang, Wen‐Chyuan Chen, Yen‐Shuo Chiu

**Affiliations:** ^1^ College of physical education Hubei Normal University Huangshi China; ^2^ Proteomics Research Center National Yang‐Ming University Taipei Taiwan; ^3^ Institute of Biochemistry and Molecular Biology National Yang‐Ming University Taipei Taiwan; ^4^ General Education Center National Taipei University of Nursing and Health Sciences Taipei Taiwan; ^5^ Graduate Institute of Sports Science National Taiwan Sport University Taoyuan Taiwan; ^6^ Center for General Education Chang Gung University of Science and Technology Taoyuan Taiwan; ^7^ Department of Otorhinolaryngology‐Head and Neck Surgery Sleep Center Linkou‐Chang Gung Memorial Hospital Taoyuan Taiwan; ^8^ Department of Orthopedics Shuang Ho Hospital Taipei Medical University Taipei Taiwan; ^9^ School of Nutrition and Health Sciences College of Nutrition Taipei Medical University Taipei Taiwan

**Keywords:** butyrate‐produce bacteria, *Lactobacillus plantarum* TWK10, lipid oxidation, proteome

## Abstract

*Lactobacillus plantarum* TWK10 (LP10) is a probiotic known to improve endurance exercise performance. Here, we analyze the proteomics and metagenomic changes in a LP10 supplemented mouse model. Male ICR mice were divided into two groups (*n* = 8) to receive by oral gavage either vehicle or of LP10 for 6 weeks. Proteins changes by LP10 treatment were subjected to the Ingenuity Pathway Analysis (IPA) to provide corroborative evidence for differential regulation of molecular and cellular functions affecting metabolic processes. Fecal samples were obtained from each mouse, and the microbial community profile analyzed by pyrosequencing of the 16S rRNA genes. Of the 880 identified proteins, 25 proteins were significantly downregulated and 44 proteins were significantly upregulated in the LP10 treated compared to vehicle group. LP10 supplementation shift in the gut microbiota to butyrate‐producing members and provided from lipid oxidation since peroxisomal fatty acid oxidation in liver.

## Graphical abstract

LP10 treatment suppress energy production in mouse liver via lipid oxidation.

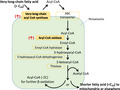

## 1 INTRODUCTION

The specie*s Lactobacillus plantarum* is a gram‐positive bacteria. Previously, we demonstrated that *Lactobacillus plantarum* TWK10 (LP10) could improve exercise performance, decrease white adipose tissue, increase muscle mass, and enhance gastrocnemius muscle type I fiber numbers without body weight gain (Chen et al.., 2016). These results suggest that gut microbiota contribute to the host metabolic phenotype to affect physical activity in terms of energy balance and body composition (Rosenbaum. , Knight, & Leibel, 2015). In this study, we investigate the mechanism by which probiotics consumption lead to changes in exercise performance and LP10’s influence on the gut microbiota to increase the efficiency of energy harvest (Murphy et al.., 2010).

Proteomics is a large‐scale comprehensive study of proteins and includes information on protein modification, along with their interacting networks (Anderson & Anderson, 1998). It is a powerful tool for studying changes in protein expression and identifying biomarkers state of a protein that correlates for pathogenic processes (Meneses‐Lorente et al.., 2006; Morand. , Macri, & Adeli, 2005; Santamaria. , Munoz, Fernandez‐Irigoyen, Prieto, & Corrales, 2007). To our knowledge, no precise mechanism has been identified to explain the increase in exercise performance with LP10 supplementation. The host metabolism, energy utilization, and storage have been revealed a tight and coordinated connection between gut microbes (Nicholson et al.., 2012). The gut microbiota was new technologies, and we are interested in how the gut microbiota regulates host expression that control metabolic processes and the energy gauge in the liver and muscle (Backhed. , Manchester, Semenkovich, & Gordon, 2007). Clinical evidence regarding the efficacy of fecal microbiota transplantation for therapy in areas including neurodevelopmental disorders, metabolic syndrome, and allergic diseases has recently emerged (Borody & Khoruts, 2011).

This current study aims to explore the effects of *L. plantarum* TWK10 (LP10) supplementation in a mouse model by shotgun proteomic analysis and fecal metagenomics analysis. We hope to identify a set of differentially expressed proteins as molecular markers for LP10 treatment and to determine how probiotic bacteria improves energy metabolism pathway (Conterno. , Fava, Viola, & Tuohy, 2011), influence efficiency of energy utilization and change microbial communities. In this study, we focus on the differential expressed proteins and uncovering the molecular mechanisms involved in LP10‐treated mice.

## 2 MATERIALS AND METHODS

### 2.1 Animals and experiment design

Male ICR mice (four weeks old) were purchased from BioLASCO (A Charles River Licensee Corp.). All mice were accommodated under maintained conditions as follows: a 12 hr light/dark cycle, room temperature kept at 24 ± 2°C, given rodent chow 5,001 and distilled water ad libitum, humidity‐controlled at 65 ± 5% conditions. Our studies were conducted in accordance with the protocols which given consent by the Institutional Ethical Committee of National Taiwan Sport University, Taoyuan City, Taiwan approved by the Institutional Animal Care and Use Committee (IACUC) (IACUC‐10405). After a two‐week acclimation period, the ICR mice (age, 6 weeks) were divided into 2 groups based on body weight (*n* = 8 per group) and received by oral gavage either vehicle or LP10 at 1.03 × 10^9^ CFU kg^‐1^ day^‐1^ for 6 weeks. The volume of vehicle or supplement administered to the vehicle group was the same, and the dose was determined according to the body weight of each mouse.

### 2.2 Determination of blood biochemical variables

At the end of the experimental period, all mice were sacrificed by 95% CO_2_ asphyxiation. Blood was immediately collected and serum separated by centrifugation. Levels of the clinical biochemical variables including aspartate aminotransferase (AST), alanine transaminase (ALT), and lactic dehydrogenase (LDH) were measured by using an autoanalyzer (Hitachi 7060).

### 2.3 Tissue glycogen determination

At the end of the experimental period, glycogen content of liver and muscles were analyzed. The method of glycogen analysis was according to a previously described method (Chen et al.., 2019).

### 2.4 Protein sample preparation for proteomic study

Liver tissue was isolated from the vehicle‐ and LP10‐treated mice. Using a Homogenizer Bullet blender (Next Advance), liver tissue was homogenized in lysis buffer containing 50 mmol/L Tris pH 6.8, 0.01% SDS, 0.01% protease inhibitor cocktail (Roche), and 0.01% phosphatase inhibitor cocktail (Sigma‐Aldrich). After 2‐min homogenization, tissue lysate was centrifuged at 4200 *g* for 10 min and supernatant was isolated by another round of centrifugation at 400 *g*, 10 min. Following centrifugation, the supernatant was aliquoted and stored at −80°C until further use.

### 2.5 SDS‐PAGE and In‐Gel Digestion

The method was modified as previous (Liao et al.., 2015), protein (50 µg) from each sample was resolved by SDS‐PAGE and the gel stained with Coomassie Brilliant Blue G‐250 (Bio‐Rad). After staining, the gel was cut into 10 slices equally and destained with 25 mmol/L NH_4_HCO_3_ ‐ 50% (v/v) acetonitrile. Slices were dried in a speed vac (Thermo Fisher Scientific), and 1% β‐mercaptoethanol and 5% 4‐vinylpyridine added for reduction of disulfide bridge and alkylation of cysteine residues. Subsequently, gel pieces were washed with 25 mmol/L NH_4_HCO_3_ and 25 mmol/L NH_4_HCO_3_ – 50% (v/v) for several times to remove the reducing and alkylating agent. The gel pieces were dried in a speed vac before incubation with a modified trypsin. The tryptic digestion was performed at 37°C. After an overnight incubation, the digest was collected and dried by speed vac. These dried peptides were kept at −20°C until further analysis. Before LC‐MS/MS analysis, the peptides were resuspended in 0.1% formic acid.

### 2.6 Nanoflow ultra high‐performance liquid chromatography − tandem mass spectrometry (nUPLC − MS/MS)

The tryptic peptides were analyzed by a nanoflow high‐performance liquid chromatography system (Agilent Technologies 1200 series) coupled to an LTQ‐Orbitrap Discovery hybrid mass spectrometer with a nanospray ionization source (Thermo Fisher Scientific). The HPLC system is equipped with LC packing C18 PepMap100 (length: 5 mm, internal diameter: 300 μm, bead size: 5 μm) as the trap column and Agilent ZORBAX XDB‐C18 (length, 50 mm; internal diameter, 75 μm; bead size 3.5 μm) as the analytical column. The mobile phase consisted of (A) 0.1% formic acid in water and (B) 0.1% formic acid in acetonitrile. For gradient elution, LC system was programmed as a 30‐min linear gradient of 5%–35% solvent followed by 95% solvent B for a duration of 10 min. For the settings of LTQ‐Orbitrap, full scans with Orbitrap analyses were collected in the range of 200–2,000 m/z. The dynamic exclusion function in the data dependent settings was activated, with the repeat count as 1, exclusion duration as 180 s, and exclusion list size as 50. Charge state rejection was activated and only charge 2 and charge 3 ions were not rejected. The top five ions in the survey scan fulfilling the above criteria were examined for their MS/MS that was generated by collision induced dissociation (CID) with the LTQ mass analyzer.

### 2.7 Database search of proteomic study

File Converter in Xcalibur 2.0.7 (Thermo Fisher Scientific) was used to extract the MS/MS information. Tandem mass spectra were interpreted using *TurboSequest* and UniProt mice database with the following parameters: fixed modifications of cysteine by vinylpyridine and variable modifications of methionine oxidation, peptide mass tolerance as 3.5 Da and fragment ion tolerance as 1.0 Da. Xcorr was used for a match with 2.5 doubly and triply charged ions. Peptides whose mass difference of parent ions were less than 10 ppm and whose Xcorr fulfilled the above requirement were considered a matched peptides. The proteins were identified when more than two peptides that met the criteria were detected from a single protein.

### 2.8 Bacterial DNA extraction and 16S rRNA sequencing

The method was modified as previous (Hsu et al., 2018). Fecal samples were collected on at the end of experiments. The collected samples were immediately stored at −80°C for DNA extraction. DNA was extracted from stool specimens using the QIAamp DNA Stool Mini Kit (Qiagen). Universal primers for the 16S variable regions V3‐5 were used for PCR amplification. The V5‐926 reverse primer included a unique sequence tag to barcode each sample. The primers used are as follows: V3‐357:5’‐CCTATCCCCTGTGTGCCTTGGCAGTCTCAGCCTACGGGAGGCAGCAG‐3’; V5‐926R: 5’‐CCATCTCATCCCTGCGTGTCTCCGACTCAGNNNNNNCCGTCAATTCMTTTRAGT‐3’. The underlined sequences denote the 454 FLX sequencing primers and the bold letters denote the universal 16S rRNA primers. The regions of 16S rRNA gene were amplified using FastStart HiFi Polymerase (Roche). Reactions were carried out on a 9700 thermal cycler (Applied Biosystems) using the following cycling parameters: 3 min at 94°C, 40 cycles of 15 s at 94°C, 45 s at 50°C, 1 min at 72°C, 72°C for 8 min and a final hold at 4°C. The presence of amplicons was confirmed by gel electrophoresis on a 1.5% agarose gel. The PCR amplicons were purified using the Agencourt AMPure XP Reagent (Beckman Coulter) and quantified on the Agilent Bioanalyzer. Equimolar amounts of the PCR amplicons were mixed in a single tube. The purified amplicon mixtures were sequenced by 454 GS Junior System using protocols recommended by the manufacturer. The sequences were analyzed by RDP Naive Bayesian rRNA Classifier Version 2.5.

### 2.9 Statistical analysis

All data are expressed mean ± *SEM* and analyzed by *t* test. A *p*‐value < .05 is considered statistically significant.

## 3 RESULTS

### 3.1 Effect of LP10 on AST, ALT and LDH, hepatic and muscle glycogen levels

**Figure 1:**
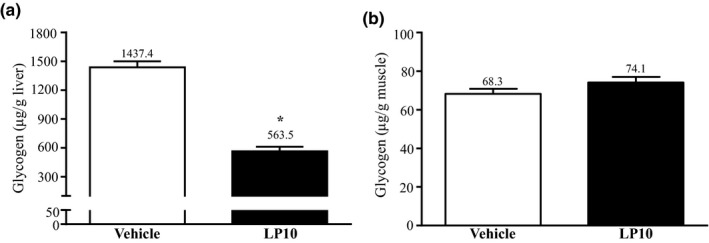
Effect of LP10 on (a) liver and (b) muscle glycogen levels at rest. Mice were pretreated with either vehicle or 1.03 × 10^9^ CFU kg^‐1^ day^‐1^ of LP10 for 6 weeks. All mice were sacrificed and examined for glycogen levels in muscle and liver tissues 1 hr after the final treatment. Data are expressed as mean ± *SEM* with *n* = 8 mice in each group. * p < .05

As compared with the vehicle group, the LP10 group shows no change in AST levels. Compared to the vehicle group, the serum ALT level of the LP10‐treated group was lower by 17.08% (*p* = .0338) and the serum LDH level lower by 18.09% (*p* = .0050). Serum concentrations of ALT, AST, and LDH are well‐recognized clinical markers of liver damage (Ferolla. , Armiliato, Couto, & Ferrari, 2015). In the present study, probiotics supplementation could have protected the activity of liver functions (data not shown). Figure 1a,b shows the glycogen content of the liver and muscle tissues in the vehicle and LP10‐treated mouse groups. The liver glycogen level in the vehicle and LP10 groups were 1,437 ± 63 and 563 ± 48 μg/g liver, respectively, with the liver glycogen content of LP10‐treated mice significantly lower by 60.80% (*p* < .0001) than the vehicle group (Figure 1a). The muscle glycogen level in vehicle and LP10‐treated groups were not significantly different at 68 ± 3 and 74 ± 3 μg/g muscle, respectively (Figure 1b).

### 3.2 Proteomic analysis

**Figure 2:**
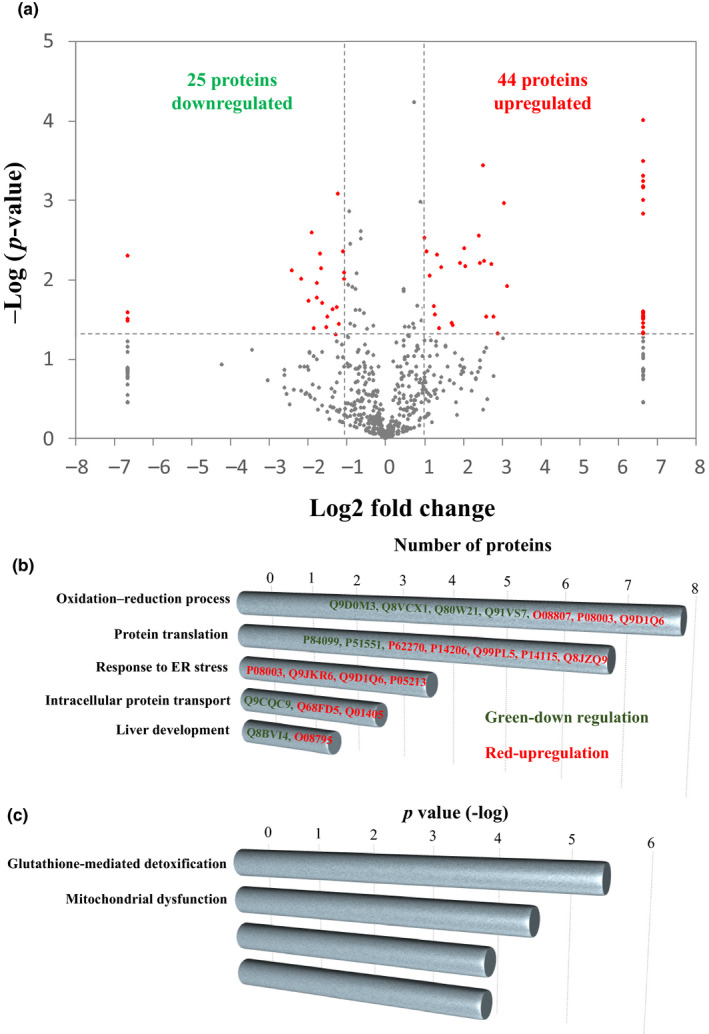
Quantitative analysis of liver proteome from mice treated with or without LP10. (a) Log ratios of label‐free quantification (LFQ) intensities in LP10‐treated mice versus control were plotted against negative log *p*‐values from Student's *t* test based on biological triplicates. Horizontal lines indicate fold changes of ±2, and vertical lines indicate *Student's t test p‐*values. Red dots denote proteins that meet both criteria for significant change between LP10‐treated and control mice. (*i.e.,* fold change in abundance >2 and *p* < .05). Gray dots denote proteins that do not fulfill these criteria. Gene Ontology (GO) and Ingenuity Pathway Analysis (IPA) of differentially expressed proteins. (b) GO term enrichment in the category “biological process” is shown. Length of bar represents the number of proteins with respective GO term. (c) Canonical pathways analysis by IPA shown. Bars indicate p‐value of relative pathways

In order to uncover the molecular changes due to probiotics supplementation, we applied a proteomic approach to analyze the liver tissue, a major metabolic organ in the body. Liver tissues collected from mice with or without LP10 treatment were lysed and separated by SDS‐PAGE. Proteins in the gel were digested by trypsin and analyzed by high‐resolution tandem mass spectrometry. The raw files were further interpreted by TurboSEQUEST. We identified a total of 880 proteins from the liver lysates in all the mouse groups. Using a label‐free quantification (LFQ) approach, we compared the liver proteins between the control and LP10 treated mice. After statistical analysis, 69 proteins were shown to have a significant change of greater than twofold (Figure 2a). For these proteins showing differential expression, 25 proteins showed a decrease and 44 proteins showed an increase in the LP10‐treated mice (Tables 1 and 2). Our results show that LP10 treatment is sufficient to alter liver proteome significantly. Details of peptide and other related information for each proteome analysis can be found in Table S1 (Supplementary data).

**Table 1:** List of 25 proteins found to be downregulated from LP10‐treated mouse liver

Description	Protein‐ID	Abbrev.	Vehicle	LP10	*p*‐value	Fold
Coiled‐coil domain‐containing protein 38	Q8CDN8	CCD38	6.435 ± 2.994	0 ± 0	.005	Vehicle only
E3 ubiquitin‐protein ligase TTC3	O88196	TTC3	0.964 ± 0.658	0 ± 0	.026	Vehicle only
Peroxisomal 2,4‐dienoyl‐CoA reductase	Q9WV68	DECR2	2.879 ± 2.064	0 ± 0	.032	Vehicle only
Dehydrogenase/reductase SDR family member 1	Q99L04	DHRS1	1.529 ± 1.097	0 ± 0	.032	Vehicle only
Cytochrome P450 1A2	P00186	CP1A2	1.065 ± 0.776	0 ± 0	.034	Vehicle only
Indolethylamine *N*‐methyltransferase	P40936	INMT	12.416 ± 3.221	2.306 ± 4.029	.008	−5.4
ATP synthase subunit g, mitochondrial	Q9CPQ8	ATP5L	2.351 ± 0.772	0.518 ± 0.615	.010	−4.5
GTP‐binding protein SAR1b	Q9CQC9	SAR1B	2.951 ± 1.078	0.745 ± 0.862	.019	−4.0
Cytochrome c oxidase subunit 2	P00405	COX2	4.737 ± 1.362	1.259 ± 0.351	.003	−3.8
60S ribosomal protein L19	P84099	RL19	1.647 ± 0.165	0.454 ± 0.908	.041	−3.6
Dihydropteridine reductase	Q8BVI4	DHPR	4.558 ± 1.663	1.32 ± 1.085	.017	−3.5
Cytochrome c1, heme protein, mitochondrial	Q9D0M3	CY1	4.088 ± 0.637	1.187 ± 1.47	.011	−3.4
S‐methylmethionine‐‐homocysteine S‐methyltransferase BHMT2	Q91WS4	BHMT2	9.694 ± 2.575	2.985 ± 1.682	.005	−3.2
Delta‐aminolevulinic acid dehydratase	P10518	HEM2	6.822 ± 0.816	2.151 ± 2.207	.007	−3.2
Glutathione S‐transferase Mu 2	P15626	GSTM2	15.768 ± 3.492	5.094 ± 5.792	.020	−3.1
Glutathione S‐transferase Mu 3	P19639	GSTM4	15.313 ± 5.83	5.269 ± 4.997	.040	−2.9
Glutathione S‐transferase Mu 7	Q80W21	GSTM7	16.539 ± 6.671	5.833 ± 3.462	.029	−2.8
UPF0585 protein C16orf13 homolog	Q9DCS2	CP013	3.656 ± 1.047	1.417 ± 1.061	.024	−2.6
Ester hydrolase C11orf54 homolog	Q91V76	CK054	3.66 ± 1.392	1.487 ± 1.087	.049	−2.5
ADP/ATP translocase 2	P51881	ADT2	9.375 ± 3.086	3.887 ± 1.831	.022	−2.4
Regucalcin	Q64374	RGN	43.571 ± 6.309	18.642 ± 5.031	.001	−2.3
Cytochrome c oxidase subunit 4 isoform 1, mitochondrial	P19783	COX41	3.741 ± 0.904	1.631 ± 1.287	.036	−2.3
Estradiol 17 beta‐dehydrogenase 5	P70694	DHB5	29.88 ± 5.223	13.88 ± 5.04	.005	−2.2
3‐oxo−5‐beta‐steroid 4‐dehydrogenase	Q8VCX1	AK1D1	4.735 ± 1.083	2.232 ± 0.799	.010	−2.1
Microsomal glutathione S‐transferase 1	Q91VS7	MGST1	23.9 ± 4.576	11.302 ± 4.589	.008	−2.1

**Table 2:** List of 44 proteins found to be upregulated in LP10‐treated mouse liver

Description	Protein‐ID	Abbrev.	Vehicle	LP10	*p*‐value	Fold
Tubulin alpha−3 chain	P05214	TBA3	0 ± 0	1.106 ± 0.245	.000	LP10 only
Tubulin alpha−8 chain	Q9JJZ2	TBA8	0 ± 0	1.792 ± 0.488	.000	LP10 only
Tubulin alpha−1A chain	P68369	TBA1A	0 ± 0	2.748 ± 0.809	.001	LP10 only
Tubulin alpha−1B chain	P05213	TBA1B	0 ± 0	4.621 ± 1.399	.001	LP10 only
Tubulin alpha−1C chain	P68373	TBA1C	0 ± 0	4.187 ± 1.304	.001	LP10 only
Fatty acid amide hydrolase 1	O08914	FAAH1	0 ± 0	1.492 ± 0.467	.001	LP10 only
Endoplasmic reticulum resident protein 44	Q9D1Q6	ERP44	0 ± 0	1.77 ± 0.594	.001	LP10 only
Neutral alpha‐glucosidase AB	Q8BHN3	GANAB	0 ± 0	2.834 ± 1.024	.001	LP10 only
Ribosome‐binding protein 1	Q99PL5	RRBP1	0 ± 0	5.17 ± 3.488	.025	LP10 only
Transcriptional activator Myb	P06876	MYB	0 ± 0	0.951 ± 0.647	.026	LP10 only
60S ribosomal protein L27a	P14115	RL27A	0 ± 0	1.461 ± 1.005	.027	LP10 only
Dipeptidyl peptidase 3	Q99KK7	DPP3	0 ± 0	0.813 ± 0.566	.028	LP10 only
Annexin A5	P48036	ANXA5	0 ± 0	0.75 ± 0.522	.028	LP10 only
Phosphomannomutase 2	Q9Z2M7	PMM2	0 ± 0	1.042 ± 0.733	.029	LP10 only
ES1 protein homolog, mitochondrial	Q9D172	ES1	0 ± 0	0.895 ± 0.635	.030	LP10 only
Peroxisomal acyl‐coenzyme A oxidase 2	Q9QXD1	ACOX2	0 ± 0	1.174 ± 0.838	.031	LP10 only
Eukaryotic translation initiation factor 3 subunit B	Q8JZQ9	EIF3B	0 ± 0	0.947 ± 0.679	.032	LP10 only
Major vault protein	Q9EQK5	MVP	0 ± 0	1.617 ± 1.198	.036	LP10 only
Valine‐‐tRNA ligase	Q9Z1Q9	SYVC	0 ± 0	1.855 ± 1.42	.040	LP10 only
2‐oxoglutarate dehydrogenase, mitochondrial	Q60597	ODO1	0 ± 0	1.586 ± 1.264	.046	LP10 only
Glucosidase 2 subunit beta	O08795	GLU2B	0 ± 0	1.188 ± 0.958	.048	LP10 only
Corticosteroid 11‐beta‐dehydrogenase isozyme 1	P50172	DHI1	0.244 ± 0.488	2.143 ± 0.955	.012	8.8
Calnexin	P35564	CALX	1.264 ± 1.705	10.396 ± 2.605	.001	8.2
Fibrinogen gamma chain	Q8VCM7	FIBG	0.277 ± 0.555	2.059 ± 1.333	.049	7.4
Protein transport protein Sec23A	Q01405	SC23A	0.208 ± 0.416	1.421 ± 0.748	.030	6.8
Very‐long‐chain acyl‐CoA synthetase	O35488	S27A2	1.387 ± 2.775	9.229 ± 2.655	.006	6.7
Talin−1	P26039	TLN1	0.416 ± 0.832	2.504 ± 1.212	.030	6.0
Zinc finger protein 76	Q8BMU0	ZNF76	1.747 ± 1.508	10.194 ± 3.745	.006	5.8
Hypoxia upregulated protein 1	Q9JKR6	HYOU1	3.264 ± 3.626	18.64 ± 2.278	.000	5.7
Peroxiredoxin−4	O08807	PRDX4	0.208 ± 0.416	1.112 ± 0.142	.006	5.3
UDP‐glucuronosyltransferase 2A3	Q8BWQ1	UD2A3	1.23 ± 1.038	6.532 ± 1.917	.003	5.3
Treslin	Q8BQ33	TICRR	1.203 ± 1.397	4.999 ± 1.251	.007	4.2
Interleukin−17B	Q9QXT6	IL17B	3.509 ± 2.556	14.342 ± 4.062	.004	4.1
40S ribosomal protein SA	P14206	RSSA	0.95 ± 1.2	3.56 ± 0.4	.006	3.7
Heat shock 70 kDa protein 1‐like	P16627	HS71L	2.269 ± 1.581	7.511 ± 3.602	.037	3.3
Radixin	P26043	RADI	0.809 ± 0.954	2.646 ± 0.968	.035	3.3
Protein disulfide‐isomerase A4	P08003	PDIA4	8.174 ± 3.08	21.947 ± 6.137	.007	2.7
Proteasome subunit beta type−3	Q9R1P1	PSB3	0.684 ± 0.818	1.776 ± 0.191	.041	2.6
Aconitate hydratase, mitochondrial	Q99KI0	ACON	8.644 ± 4.446	21.438 ± 3.868	.005	2.5
Acyl‐CoA synthetase family member 2, mitochondrial	Q8VCW8	ACSF2	5.628 ± 4.992	13.464 ± 2.112	.028	2.4
40S ribosomal protein S18	P62270	RS18	1.305 ± 1.035	3.105 ± 0.536	.021	2.4
Clathrin heavy chain 1	Q68FD5	CLH1	5.679 ± 2.545	12.41 ± 2.468	.009	2.2
Dimethylglycine dehydrogenase, mitochondrial	Q9DBT9	M2GD	19.149 ± 6.913	39.658 ± 6.145	.004	2.1
Microsomal triglyceride transfer protein large subunit	O08601	MTP	8.217 ± 2.892	16.567 ± 1.936	.003	2.0

### 3.3 Gene ontology and ingenuity pathways analysis

The hepatic proteins whose expressions are changed by LP10 treatment were analyzed by their Gene Ontology (GO) and canonical signaling pathways (Figure 2b,c). In Figure 2b, proteins downregulated by LP10‐treatment are highlighted in green while those that are upregulated are highlighted in red. Eight proteins were shown to be involved in the oxidation–reduction pathway, with five proteins being downregulated and three proteins upregulated. Seven proteins were involved in protein analysis (two proteins downregulated and five proteins upregulated), four proteins upregulated that are involved in ER stress response, three proteins involved in intracellular protein transport (1 downregulated and 2 upregulated), and two proteins involved in liver development (1 downregulated and 2 upregulated). Figure 2c shows the major pathways generated by IPA between the vehicle and LP10‐treated groups using a threshold *p*‐value < .05. The length of the bar indicates only that the differentially expressed proteins are related to this pathway but does not indicate direction of regulation. The proteins regulated by LP10 are involved in glutathione‐mediated detoxification, mitochondrial dysfunction, remodeling of epithelial adherens junctions, and LPS/IL‐1‐mediated inhibition of RXR function. Both the GO and IPA analyses show that the differentially expressed proteins are involved in the oxidation–reduction process. The glutathione‐mediated detoxification pathway gave a *p*‐value of −log 5.6, indicating that this pathway was significantly changed by LP10 treatment.

### 3.4 Taxonomic shifts due to LP10 supplementation

Gene classifier assigned the usable raw reads to 7 phyla, 11 classes, 18 families, and 42 genus. The most abundant phyla include the *Bacteroidetes* and *Firmicutes*, classes include *Bacteroidia* and *Clostridia,* families include *Lachnospiraceae*, *Porphyromonadaceae, Rikenellaceae,* and *Ruminococcaceae,* and genus include *Alistipes*, *Barnesiella,* and *Odoribacter* (Table 3). Our data demonstrate that LP10 supplementation may dramatically impact microbial taxonomy when compared with the vehicle group. The relative abundance of the *Bacteroidetes* phyla was significantly decreased by 17.28% (*p* = .011) and *Firmicutes* phyla significantly increased by 2.55‐fold (*p* = .0093) with LP10 treatment (Table 3). At the class level, the relative abundance of *Bacteroidia* in LP10 treatment was significantly decreased by 21.24% *(p* = .0024) and *Clostridia* significantly increased by 3.63‐fold (*p* = .0016) compared with the vehicle group. At the families level, the relative abundance of *Rikenellaceae* with LP10 treatment was significantly decreased by 78.37% (*p* = .0028) and *Porphyromonadaceae* significantly increased by 2.42‐fold (*p* = .0035) compared with the vehicle group. At the genera level, *Alistipes* was significantly decrease by 23.96% (*p* = .0258) and *Barnesiella* significantly increased by 9.43‐fold (*p* = .0415) with LP10 treatment.

**Table 3:** Predominant fecal bacteria phyla, order, family, and genus present in vehicle or LP10‐supplemented mice

Phylum	Class	Family	Genes	Vehicle	LP10	*p*‐values
Relative abundance %						
*Bacteroidetes*				86.90 ± 2.95	71.88 ± 1.57	.0110
	*Bacteroidales*			89.98 ± 2.55	70.87 ± 1.11	.0024
		*Porphyromonadaceae*		23.18 ± 2.10	56.14 ± 4.90	.0035
			*Barnesiella*	2.73 ± 0.62	25.73 ± 7.73	.0415
		*Rikenellaceae*		65.76 ± 1.88	14.22 ± 3.91	.0028
		*no detect*	*Alistipes*	90.64 ± 1.97	68.93 ± 5.94	.0258
		*no detect*	*Odoribacter*	6.63 ± 2.15	5.34 ± 2.84	.7391
*Firmicutes*				15.01 ± 3.99	38.32 ± 2.92	.0093
	*Clostridiales*			11.05 ± 3.13	40.16 ± 2.18	.0016
		*Ruminococcaceae*		1.62 ± 0.45	1.10 ± 0.38	.4210
		*Lachnospraceae*		9.44 ± 3.64	23.40 ± 5.59	.0748

## 4 DISCUSSION

LP10 appears to enhance the reducing power of liver cells as observed by our list of differentially expressed proteins. For instance, peroxiredoxin, an enzyme that promotes reduction of reactive oxygen species, showed increased levels in the liver after LP10 treatment. On the other hand, we observed downregulation of glutathione S‐transferase, an enzyme that facilitates glutathione oxidation. These results suggest that the liver cells undergo some oxidative reaction upon LP10 treatment, altering the proteins with reducing power to maintain redox equilibrium. In support of this, we found the upregulation of several proteins required for fatty acid oxidation. Peroxisomal acyl‐coenzyme A oxidase 2 (ACOX2) and very‐long‐chain acyl‐CoA synthetase (S27A2) were significantly increased in LP10‐treated mice. These proteins are required for processing/metabolism/catabolism of very‐long‐chain fatty acids (VLCFAs), *for example,* lignocerate. VLCFAs must be shortened by peroxisomal beta‐oxidation before they can be catabolized in the mitochondria.

The first step of VLCFAs breakdown to convert VLCFAs to very‐long‐chain acyl CoA requires the very‐long‐chain acyl‐CoA synthetase (Wanders, 2014). We observed a 6.7‐fold increase in the expression of very‐long‐chain acyl‐CoA synthetase in LP10‐treated mice. For peroxisomal acyl‐coenzyme A oxidase 2, the second enzyme that oxidizes VLCFAs in peroxisome, it was detected only in the LP10‐treated mice, and not the vehicle‐treated mice. These results are consistent with the notion that the rate‐determining steps for a biochemical reaction are usually within the first few steps. Fatty acid oxidation is likely the main cause of changing redox balance in the livers of LP10‐treated mice. Normally, after VLCFAs are shortened by peroxisomal beta‐oxidation, the shorter fatty acids may be transported into mitochondria for further catabolism. One of the final products of beta‐oxidation is acetyl CoA, which is used to generate ATP through the TCA cycle and oxidative phosphorylation. We did not observe any changes in the mitochondria‐derived fatty acid oxidation enzymes. However, we found that level of ATP synthase subunit g (ATP5L) and cytochrome c oxidase subunit 2 (COX2) were downregulated by 4.5‐ and 3.8‐fold, respectively, in LP10‐treated mice. These proteins are reported to be essential for maximal levels of respiration, ATP synthesis and cytochrome c oxidase activity in yeast (Wanders, 2014). Our data support the idea that LP10 treatment may suppress energy production in mouse liver.

In our previous (Chen et al.., 2016) and present study, we did not see any obvious liver steatosis or liver weight gain in LP10‐treated mice, although we observed a reduction in liver glycogen levels and muscle hypertrophy. These observations taken together with the proteomic data suggest that the catabolized fatty acids in liver may be transported to other organs such as the muscles. In our list or upregulated proteins, two proteins were identified that are involved in fatty acid transportation–microsomal triglyceride transfer protein large subunit (MTP) and protein disulfide‐isomerase A4 (PDIA4). MTP forms a heterodimer with PDI to play a necessary role of biosynthesis of apolipoprotein B (apoB)‐containing triglyceride‐rich lipoproteins. MTP and PDIA4 increased 2‐ and 2.7‐fold, respectively, in LP10‐treated mice. Similar stoichiometric changes of these proteins support the model that the activity of fatty acid transportation is controlled by the complex formation of MTP and PDIA4 (Wetterau. , Aggerbeck, Laplaud, & McLean, 1991). The proteome data suggest that the peroxisomal fatty acid oxidation in liver provides energy source to other organs, rather than to the liver itself.

Although the *Lactobacillus* population did not appear as one of the classified genus listing, indicating that LP10 did not colonize in mice cecum, the intestinal microbiota did dramatically shift a result of LP10 supplementation. The effect of endurance exercise performance and physiological adaptations of is through nitrosative stress and modulation of oxidative and as a way to avoid bacterial translocation, tissue damage, and intestinal permeability (Bessa et al.., 2016; Lamprecht & Frauwallner, 2012). The shifting of colonic microbiota to increase the abundance of *Firmicutes* phylum could activate electrogenic Cl^−^ channels in the intestinal mucosa secretion, which functions as a primitive innate defense mechanism (Musch. , Wang, Claud, & Chang, 2013). This defense mechanism plays important role in protecting intestinal infection by inducing pro‐inflammatory and pro‐oxidant responses that control pathogen load, helping to prevent fatal dehydration (Ghosh et al.., 2011). Thus, the *Firmicutes* phyla that is increased with LP10 supplementation could improve endurance exercise performance by benefitting the gut microbiota. Other studies have documented beneficial effects of probiotic and prebiotics interventions through alterations of *Clostridia* (Lin. , Chen, Tsai, & Pan, 2017; Onrust et al.., 2015).

These results observations are both intriguing and slightly contrary to previous research. In general, lower *Bacteroidetes* and greater *Firmicutes* are sometimes observed in detrimental metabolic states such as obesity and diabetes (Geurts et al.., 2011; Ley. , Turnbaugh, Klein, & Gordon, 2006; Zhang et al.., 2013). *Clostridia* is recognized as a species responsible for several human and animal diseases (Burke & Lamont, 2014). Clarke *et al*. compared the fecal bacterial profile of male elite rugby players to healthy subjects and found that athletes had lowered *Bacteroidetes* and increased *Firmicutes*, similar to our observations in LP10 supplementation mice (Clarke et al.., 2014). The *Firmicutes* phyla has butyrate as its primary metabolic end product (Macfarlane & Macfarlane, 2003) and recent studies in rats have demonstrated that exercise is associated with greater butyrate levels (Matsumoto et al.., 2008), exerting multiple beneficial effects on mammalian energy metabolism (den Besten et al.., 2013). In addition, *Clostridia* also has butyrate‐producing capacity to reduce lipogenesis (Van den Abbeele et al.., 2013; Zhao. , Guo, Liu, Gao, & Nie, 2014). Therefore, elevations in butyrate‐producing members of the *Firmicutes* and *Clostridia* phyla may actually be advantageous to endurance exercise performance.

In conclusion, the liver is an important metabolic organ. Liver receives the hormonal signals and existing energy stores and program the multiple processes of energy storage such as gluconeogenesis, glycogenesis, lipogenesis, and mobilization of energy supplies for body and the rest of the processes as β‐oxidation and ketogenesis glycolysis and glycogenolysis (Youngson. , Morris, & Ballard, 2017).

The liver–gut axis is interesting, they have close functional and bidirectional communication between of liver and gut, and the changes of specific intestinal microbiota are characteristic of general obesogenic and dysmetabolic hepatic lipogenesis, and typical of the first stage of non‐alcoholic fatty liver disease (NAFLD) (Backhed et al.., 2004). This is evidenced from obesity studies showing that specific gut microbiota (e.g., *Bacteroides thetaiotaomicron*) become the dominant bacteria for more efficient intestinal absorption of increased of lipid deposition, absorbing more calories and poorly digestible plant polysaccharides (Backhed et al.., 2004). VLCFAs are the first lipid to activated in the cytosol to long‐chain acyl‐CoAs (coenzyme A) via long‐chain acyl‐CoA synthetases (Lee et al.., 2017). LP10 supplementation promotes the peroxisomal fatty acid oxidation in liver to provide energy to muscles. The gut microbiota transfers to the butyrate‐producing members to increase host fatty acid utilization.

It is known that energy utilization is correlated with the type of exercise. Long‐distance runners preferentially use fat as energy source and positively correlated with fat utilization compared with short‐distance runners (Brooks. & Mercier 1994 ). In previous study, we observed that LP10‐treated mice increased their muscular endurance in several behavioral tests (Chen et al.., 2016). In conclusion, our proteomic and metagenomic analyses provide evidence for alteration of energy utilization in liver. LP10 supplementation causes the host fatty acid transported via bloodstream to be taken up by organs, including the muscle, where they are further metabolized in the mitochondria to generate energy.

## CONFLICT OF INTEREST

The authors declare no conflict of interest.

## AUTHOR CONTRIBUTIONS

Yi‐Ming Chen, Chen‐Chung Liao, Chi‐Chang Huang and Yen‐Shuo, Chiu designed the experiments; Yi‐Ming Chen, Chen‐Chung Liao, Yen‐Chun Huang, and Ming‐Yi Chen carried out the laboratory experiments; Yi‐Ming Chen, Yen‐Chun Huang, Wen‐Chyuan Chen, Chi‐Chang Huang and Yen‐Shuo, Chiu analyzed the data, interpreted the results, prepared figures, and wrote the manuscript; Chen‐Chung Liao, Wen‐Chyuan Chen, and Yen‐Shuo, Chiu contributed reagents, materials, and analysis platforms and revised manuscript.

## Supporting information

Table S1
