# Supplementary material for: Proteome and microbiota analysis highlight Lactobacillus plantarum TWK10 supplementation improves energy metabolism and exercise performance in mice
Source: Food Sci Nutr. 2020 Jun 3;8(7):3525–34. doi: 10.1002/fsn3.1635 (PMC7382123; doi:10.1002/fsn3.1635)
Supplement: Supplementary file 1 — Table S1 [file FSN3-8-3525-s001.docx]

**Supplementary Information**

**Table S1.** Differential liver protein expression between vehicle and LP10-treated mice ranked by *p*-value.

| **Description** | **Protein-ID** | **Abbrev.** | **Vehicle** | | **LP10** | | ***p* value** | **Fold** |
| --- | --- | --- | --- | --- | --- | --- | --- | --- |
| 78 proteins significantly decreased in the LP10 group | | | | | | | | |
| Elongation factor 1-alpha | Q3UZQ3 | Q3UZQ3 | 18.7 ± 5.48 | | 0.76 ± 0.76 | | 0.018 | -24.74 |
| Indolethylamine N-methyltransferase | P40936 | INMT | 12.42 ±1.61 | | 2.31 ± 2.01 | | 0.008 | -5.38 |
| ATP synthase subunit g, mitochondrial | Q9CPQ8 | ATP5L | 2.35 ± 0.39 | | 0.52 ± 0.31 | | 0.010 | -4.54 |
| Putative uncharacterized protein | Q9CQY3 | Q9CQY3 | 2.35 ± 0.39 | | 0.52 ± 0.31 | | 0.010 | -4.54 |
| Putative uncharacterized protein | Q9D037 | Q9D037 | 2.35 ± 0.39 | | 0.52 ± 0.31 | | 0.010 | -4.54 |
| Putative uncharacterized protein | Q3TJZ1 | Q3TJZ1 | 6.03 ± 1.43 | | 1.37 ± 1.02 | | 0.038 | -4.4 |
| Putative uncharacterized protein | Q3TLB1 | Q3TLB1 | 6.03 ± 1.43 | | 1.37 ± 1.02 | | 0.038 | -4.4 |
| Protein 0610011F06Rik | G5E8X1 | G5E8X1 | 3.66 ± 0.52 | | 0.84 ± 0.52 | | 0.009 | -4.33 |
| GTP-binding protein SAR1b | Q9CQC9 | SAR1B | 2.95 ± 0.54 | | 0.75 ± 0.43 | | 0.019 | -3.96 |
| Putative uncharacterized protein | Q3U281 | Q3U281 | 2.95 ± 0.54 | | 0.75 ± 0.43 | | 0.019 | -3.96 |
| Cytochrome c oxidase subunit 2 | P00405 | COX2 | 4.74 ± 0.68 | | 1.26 ± 0.18 | | 0.003 | -3.76 |
| Cytochrome c oxidase subunit 2 | A0A075DC90 | A0A075DC90 | 4.74 ± 0.68 | | 1.26 ± 0.18 | | 0.003 | -3.76 |
| Cytochrome c oxidase subunit 2 | A3E4B0 | A3E4B0 | 4.74 ± 0.68 | | 1.26 ± 0.18 | | 0.003 | -3.76 |
| Cytochrome c oxidase subunit 2 | A3R481 | A3R481 | 4.74 ± 0.68 | | 1.26 ± 0.18 | | 0.003 | -3.76 |
| Cytochrome c oxidase subunit 2 | K7XK22 | K7XK22 | 4.74 ± 0.68 | | 1.26 ± 0.18 | | 0.003 | -3.76 |
| Cytochrome c oxidase subunit 2 | K7XKA7 | K7XKA7 | 4.74 ± 0.68 | | 1.26 ± 0.18 | | 0.003 | -3.76 |
| Putative uncharacterized protein | Q3TX45 | Q3TX45 | 4.86 ± 1.25 | | 1.32 ± 0.43 | | 0.037 | -3.67 |
| **Table S1.** *Cont.* | | | | | | | | |
| **Description** | **Protein-ID** | **Abbrev.** | **Vehicle** | | **LP10** | | ***p* value** | **Fold** |
| 60S ribosomal protein L19 | P84099 | RL19 | 1.65 ± 0.08 | | 0.45 ± 0.45 | | 0.041 | -3.63 |
| Adenosylhomocysteinase | Q5M9P0 | Q5M9P0 | 12.53 ±2.42 | | 3.52 ± 2.57 | | 0.043 | -3.56 |
| Dihydropteridine reductase | Q8BVI4 | DHPR | 4.56 ± 0.83 | | 1.32 ± 0.54 | | 0.017 | -3.45 |
| Cytochrome c1, heme protein, mitochondrial | Q9D0M3 | CY1 | 4.09 ± 0.32 | | 1.19 ± 0.74 | | 0.011 | -3.44 |
| S-methylmethionine--homocysteine S-methyltransferase BHMT2 | Q91WS4 | BHMT2 | 9.69 ± 1.29 | | 2.98 ± 0.84 | | 0.005 | -3.25 |
| Delta-aminolevulinic acid dehydratase | P10518 | HEM2 | 6.82 ± 0.41 | | 2.15 ± 1.1 | | 0.007 | -3.17 |
| Delta-aminolevulinic acid dehydratase | Q9DD05 | Q9DD05 | 6.82 ± 0.41 | | 2.15 ± 1.1 | | 0.007 | -3.17 |
| Glutathione S-transferase Mu 2 | P15626 | GSTM2 | 15.77 ±1.75 | | 5.09 ± 2.9 | | 0.020 | -3.1 |
| Glutathione S-transferase | Q9DCU1 | Q9DCU1 | 106.21 10.55 | | 36.02 ± 6.33 | | 0.001 | -2.95 |
| Glutathione S-transferase Mu 3 | P19639 | GSTM4 | 15.31 ±2.91 | | 5.27 ± 2.5 | | 0.040 | -2.91 |
| Glutathione S-transferase Mu 7 | Q80W21 | GSTM7 | 16.54 ±3.34 | | 5.83 ± 1.73 | | 0.029 | -2.84 |
| Glutathione S-transferase Mu 7 | D3YVP5 | D3YVP5 | 16.54 ±3.34 | | 5.83 ± 1.73 | | 0.029 | -2.84 |
| Catalase | Q3UZE7 | Q3UZE7 | 45.96 ±10.89 | | 16.67 ± 4.59 | | 0.048 | -2.76 |
| Coiled-coil domain containing 83, isoform CRA_b | D3YUZ1 | D3YUZ1 | 2.1 ± 0.23 | | 0.76 ± 0.44 | | 0.037 | -2.74 |
| Gstm7 protein | Q6PJ91 | Q6PJ91 | 20.34 ±4.15 | | 7.59 ± 1.64 | | 0.029 | -2.68 |
| UPF0585 protein C16orf13 homolog | Q9DCS2 | CP013 | 3.66 ± 0.52 | | 1.42 ± 0.53 | | 0.024 | -2.58 |
| Ester hydrolase C11orf54 homolog | Q91V76 | CK054 | 3.66 ± 0.7 | | 1.49 ± 0.54 | | 0.049 | -2.46 |
| ADP/ATP translocase 2 | P51881 | ADT2 | 9.38 ± 1.54 | | 3.89 ± 0.92 | | 0.022 | -2.41 |
| MCG14318, isoform CRA_a | Q6NSR5 | Q6NSR5 | 9.97 ± 2.07 | | 4.24 ± 0.67 | | 0.039 | -2.35 |
| Regucalcin | Q64374 | RGN | 43.57 ±3.15 | | 18.64 ± 2.52 | | 0.001 | -2.34 |
| **Table S1.** *Cont.* | | | | | | | | |
| **Description** | **Protein-ID** | **Abbrev.** | **Vehicle** | | **LP10** | | ***p* value** | **Fold** |
| Cytochrome c oxidase subunit 4 isoform 1, mitochondrial | P19783 | COX41 | 3.74 ± 0.45 | | 1.63 ± 0.64 | | 0.036 | -2.29 |
| Estradiol 17 beta-dehydrogenase | P70694 | DHB5 | 29.88 ±2.61 | 13.88 ± 2.52 | | | 0.005 | -2.15 |
| 3-oxo-5-beta-steroid 4-dehydrogenase | Q8VCX1 | AK1D1 | 4.74 ± 0.54 | 2.23 ± 0.4 | | | 0.010 | -2.12 |
| Microsomal glutathione S-transferase 1 | Q91VS7 | MGST1 | 23.9 ± 2.29 | 11.3 ± 2.29 | | | 0.008 | -2.11 |
| Microsomal glutathione S-transferase 1 | E9QJW0 | E9QJW0 | 22.09 ±3.34 | 11.3 ± 2.29 | | | 0.037 | -1.95 |
| Carbonic anhydrase 2 | P00920 | CAH2 | 3.92 ± 0.51 | 2.01 ± 0.17 | | | 0.012 | -1.95 |
| Carbonic anhydrase 3 | P16015 | CAH3 | 89.22 ±4.36 | 46.91 ± 6.2 | | | 0.001 | -1.9 |
| NADH-cytochrome b5 reductase 3 | Q9DCN2 | NB5R3 | 8.4 ± 0.61 | 4.47 ± 0.59 | | | 0.004 | -1.88 |
| Phosphoglycerate mutase 1 | Q9DBJ1 | PGAM1 | 6.58 ± 0.85 | 3.51 ± 0.77 | | | 0.036 | -1.88 |
| Putative uncharacterized protein | Q3UAA9 | Q3UAA9 | 22.23 ±2.28 | 12.07 ± 2.13 | | | 0.017 | -1.84 |
| Peroxiredoxin-1 | P35700 | PRDX1 | 18.21 ±1.99 | 9.97 ± 1.24 | | | 0.013 | -1.83 |
| Glutathione S-transferase A3 | P30115 | GSTA3 | 118.62 ±17.15 | 67.96 ± 8.84 | | | 0.039 | -1.75 |
| Adenylate kinase 2, mitochondrial | Q9WTP6 | KAD2 | 8.41 ± 0.87 | 4.89 ± 0.8 | | | 0.025 | -1.72 |
| Retinol dehydrogenase 7 | O88451 | RDH7 | 14.42 ± 1.2 | 8.39 ± 1.26 | | | 0.013 | -1.72 |
| ATP synthase subunit O, mitochondrial | Q9DB20 | ATPO | 14.58 ±1.27 | 8.65 ± 0.87 | | | 0.008 | -1.69 |
| 40S ribosomal protein S5 | Q91V55 | Q91V55 | 3.82 ± 0.24 | 2.3 ± 0.33 | | | 0.010 | -1.66 |
| 4-hydroxyphenylpyruvate dioxygenase | P49429 | HPPD | 18.96 ±1.79 | 11.93 ± 1.53 | | | 0.024 | -1.59 |
| Thiosulfate sulfurtransferase | P52196 | THTR | 15.73 ±1.02 | 10.03 ± 0.62 | | | 0.003 | -1.57 |
| Electron transfer flavoprotein subunit beta | Q9DCW4 | ETFB | 27.47 ±1.68 | 17.55 ± 1.08 | | | 0.003 | -1.57 |
| **Table S1.** *Cont* | | | | | | | | |
| **Description** | **Protein-ID** | **Abbrev.** | **Vehicle** | **LP10** | | | ***p* value** | **Fold** |
| Alcohol dehydrogenase [NADP(+)] | Q9JII6 | AK1A1 | 11.7 ± 0.9 | 7.86 ± 0.96 | | | 0.027 | -1.49 |
| Putative uncharacterized protein | Q3UJW9 | Q3UJW9 | 11.7 ± 0.9 | 7.86 ± 0.96 | | | 0.027 | -1.49 |
| Enoyl-CoA delta isomerase 1, mitochondrial | P42125 | ECI1 | 5.82 ± 0.42 | 4.15 ± 0.5 | | | 0.043 | -1.4 |
| Putative uncharacterized protein | Q9DBN7 | Q9DBN7 | 5.82 ± 0.42 | 4.15 ± 0.5 | | | 0.043 | -1.4 |
| Catalase | Q542K4 | Q542K4 | 85.94 ±4.08 | 62.14 ± 7 | | | 0.026 | -1.38 |
| 2,4-dienoyl-CoA reductase, mitochondrial | Q9CQ62 | DECR | 7.89 ± 0.5 | 6.37 ± 0.3 | | | 0.039 | -1.24 |
| Argininosuccinate lyase | E0CXM2 | E0CXM2 | 4.94 ± 1.94 | 0 ± 0 | | | 0.043 | -100 |
| Cofilin-1 | F8WGL3 | F8WGL3 | 1.89 ± 0.64 | 0 ± 0 | | | 0.026 | -100 |
| Coiled-coil domain-containing protein 38 | Q8CDN8 | CCD38 | 6.43 ± 1.5 | 0 ± 0 | | | 0.005 | -100 |
| Cytochrome P450 1A2 | P00186 | CP1A2 | 1.07 ± 0.39 | 0 ± 0 | | | 0.034 | -100 |
| Cytochrome P450 2C29 | Q3UT49 | Q3UT49 | 2.82 ± 1 | 0 ± 0 | | | 0.030 | -100 |
| Dehydrogenase/reductase SDR family member 1 | Q99L04 | DHRS1 | 1.53 ± 0.55 | 0 ± 0 | | | 0.032 | -100 |
| E3 ubiquitin-protein ligase TTC3 | O88196 | TTC3 | 0.96 ± 0.33 | 0 ± 0 | | | 0.026 | -100 |
| Histidine ammonia-lyase | Q8CE60 | Q8CE60 | 2.12 ± 0.84 | 0 ± 0 | | | 0.045 | -100 |
| Peroxisomal 2,4-dienoyl-CoA reductase | Q9WV68 | DECR2 | 2.88 ± 1.03 | 0 ± 0 | | | 0.032 | -100 |
| Protein disulfide-isomerase | Q3U738 | Q3U738 | 5.9 ± 2.39 | 0 ± 0 | | | 0.049 | -100 |
| Protein DJ-1 | A2A813 | A2A813 | 1.31 ± 0.5 | 0 ± 0 | | | 0.039 | -100 |
| Putative uncharacterized protein | Q3TR93 | Q3TR93 | 2.88 ± 1.03 | 0 ± 0 | | | 0.032 | -100 |
| Putative uncharacterized protein | Q3TVU9 | Q3TVU9 | 1.53 ± 0.55 | 0 ± 0 | | | 0.032 | -100 |
| **Table S1.** *Cont* | | | | | | | | |
| **Description** | **Protein-ID** | **Abbrev.** | **Vehicle** | **LP10** | | | ***p* value** | **Fold** |
| Putative uncharacterized protein | Q3UEF2 | Q3UEF2 | 2.82 ± 1 | 0 ± 0 | | | 0.030 | -100 |
| Putative uncharacterized protein | Q3UEY0 | Q3UEY0 | 2.88 ± 1.03 | 0 ± 0 | | | 0.032 | -100 |
| Putative uncharacterized protein | Q9CX22 | Q9CX22 | 1.89 ± 0.64 | 0 ± 0 | | | 0.026 | -100 |
| 123 proteins significantly increased in the LP10 group | | | | | | | | |
| Carbamoyl-phosphate synthase [ammonia], mitochondrial | Q8C196 | CPSM | 283.2 ± 7.89 | | | 388.5 ± 29.79 | **0.014** | 1.37 |
| Cofilin-1 | P18760 | COF1 | 2.53 ± 0.13 | | | 3.49 ± 0.24 | **0.013** | 1.38 |
| Serotransferrin | Q921I1 | TRFE | 28.69 ± 1.76 | | | 40.57 ± 4.2 | **0.040** | 1.41 |
| 40S ribosomal protein S24 | P62849 | RS24 | 2.02 ± 0.32 | | | 3.03 ± 0.22 | **0.039** | 1.5 |
| 40S ribosomal protein S24 | Q3TIF8 | Q3TIF8 | 2.02 ± 0.32 | | | 3.03 ± 0.22 | **0.039** | 1.5 |
| 40S ribosomal protein S24 | Q9CQK2 | Q9CQK2 | 2.02 ± 0.32 | | | 3.03 ± 0.22 | **0.039** | 1.5 |
| 40S ribosomal protein S24 | Q9CY61 | Q9CY61 | 2.02 ± 0.32 | | | 3.03 ± 0.22 | **0.039** | 1.5 |
| 40S ribosomal protein S24 | Q9D7P1 | Q9D7P1 | 2.02 ± 0.32 | | | 3.03 ± 0.22 | **0.039** | 1.5 |
| Acyl-coenzyme A synthetase ACSM1, mitochondrial | Q91VA0 | ACSM1 | 11.06 ± 2.48 | | | 17.96 ± 0.7 | **0.036** | 1.62 |
| 78 kDa glucose-regulated protein | P20029 | GRP78 | 32.38 ± 2.02 | | | 53.47 ± 0.56 | **0.000** | 1.65 |
| 40S ribosomal protein S3 | P62908 | RS3 | 2.55 ± 0.59 | | | 4.22 ± 0.26 | **0.041** | 1.65 |
| Putative uncharacterized protein | Q3UCL7 | Q3UCL7 | 2.55 ± 0.59 | | | 4.22 ± 0.26 | **0.041** | 1.65 |
| Putative uncharacterized protein | Q9D0A2 | Q9D0A2 | 2.55 ± 0.59 | | | 4.22 ± 0.26 | **0.041** | 1.65 |
| Glyceraldehyde-3-phosphate dehydrogenase | S4R1W1 | S4R1W1 | 12.4 ± 3.16 | | | 21.67 ± 2.07 | **0.050** | 1.75 |
| Transitional endoplasmic reticulum ATPase | Q01853 | TERA | 15.35 ± 3 | | | 27.01 ± 2.33 | **0.022** | 1.76 |
| Pyruvate carboxylase | E9QPD7 | E9QPD7 | 21.17 ± 5.15 | | | 38.74 ± 2.36 | **0.021** | 1.83 |
| Pyruvate carboxylase | G5E8R3 | G5E8R3 | 21.17 ± 5.15 | | | 38.74 ± 2.36 | **0.021** | 1.83 |
| Pyruvate carboxylase | Q3T9S7 | Q3T9S7 | 21.17 ± 5.15 | | | 38.74 ± 2.36 | **0.021** | 1.83 |
| Pyruvate carboxylase | Q3TCQ3 | Q3TCQ3 | 21.17 ± 5.15 | | | 38.74 ± 2.36 | **0.021** | 1.83 |
| MCG1788 | Q3UEP4 | Q3UEP4 | 12.35 ± 2.17 | | | 22.75 ± 1.27 | **0.006** | 1.84 |
| Calreticulin | P14211 | CALR | 13.78 ± 1.5 | | | 25.79 ± 1.39 | **0.001** | 1.87 |
| **Table S1.** *Cont* | | | | | | | | |
| **Description** | **Protein-ID** | **Abbrev.** | **Vehicle** | | | **LP10** | ***p* value** | **Fold** |
| UDP-glucuronosyltransferase 2B17 | P17717 | UDB17 | 12.96 ± 3.34 | | | 24.45 ± 2.48 | **0.033** | 1.89 |
| Putative uncharacterized protein | Q3UBL9 | Q3UBL9 | 12.89 ± 1.28 | | | 24.43 ± 1.09 | **0.000** | 1.9 |
| L-lactate dehydrogenase | Q3THB4 | Q3THB4 | 9.65 ± 2.28 | | | 18.4 ± 0.86 | **0.011** | 1.91 |
| Protein disulfide-isomerase A6 | Q922R8 | PDIA6 | 8.02 ± 0.79 | | | 15.63 ± 2.39 | **0.023** | 1.95 |
| Endoplasmin | P08113 | ENPL | 28.64 ± 2.97 | | | 56.63 ± 3.76 | **0.001** | 1.98 |
| Heat shock protein 90, beta (Grp94), member 1 | Q91V38 | Q91V38 | 28.64 ± 2.97 | | | 56.63 ± 3.76 | **0.001** | 1.98 |
| Putative uncharacterized protein | Q3UBU0 | Q3UBU0 | 28.64 ± 2.97 | | | 56.63 ± 3.76 | **0.001** | 1.98 |
| Microsomal triglyceride transfer protein large subunit | O08601 | MTP | 8.22 ± 1.45 | | | 16.57 ± 0.97 | **0.003** | 2.02 |
| Dimethylglycine dehydrogenase, mitochondrial | Q9DBT9 | M2GD | 19.15 ± 3.46 | | | 39.66 ± 3.07 | **0.004** | 2.07 |
| Putative uncharacterized protein | Q3TF16 | Q3TF16 | 8.14 ± 2.86 | | | 17.17 ± 1.03 | **0.025** | 2.11 |
| Clathrin heavy chain | Q5SXR6 | Q5SXR6 | 5.68 ± 1.27 | | | 12.41 ± 1.23 | **0.009** | 2.19 |
| Clathrin heavy chain 1 | Q68FD5 | CLH1 | 5.68 ± 1.27 | | | 12.41 ± 1.23 | **0.009** | 2.19 |
| 40S ribosomal protein S18 | P62270 | RS18 | 1.3 ± 0.52 | | | 3.11 ± 0.27 | **0.021** | 2.38 |
| Acyl-CoA synthetase family member 2, mitochondrial | Q8VCW8 | ACSF2 | 5.63 ± 2.5 | | | 13.46 ± 1.06 | **0.028** | 2.39 |
| Aconitate hydratase, mitochondrial | Q99KI0 | ACON | 8.64 ± 2.22 | | | 21.44 ± 1.93 | **0.005** | 2.48 |
| Proteasome subunit beta type-3 | Q9R1P1 | PSB3 | 0.68 ± 0.41 | | | 1.78 ± 0.1 | **0.041** | 2.6 |
| Protein disulfide-isomerase A4 | P08003 | PDIA4 | 8.17 ± 1.54 | | | 21.95 ± 3.07 | **0.007** | 2.69 |
| Putative uncharacterized protein | Q3THL7 | Q3THL7 | 1 ± 0.62 | | | 2.98 ± 0.42 | **0.038** | 2.98 |
| Putative uncharacterized protein | Q3TIG8 | Q3TIG8 | 1 ± 0.62 | | | 2.98 ± 0.42 | **0.038** | 2.98 |
| Radixin | P26043 | RADI | 0.81 ± 0.48 | | | 2.65 ± 0.48 | **0.035** | 3.27 |
| Heat shock 70 kDa protein 1-like | P16627 | HS71L | 2.27 ± 0.79 | | | 7.51 ± 1.8 | **0.037** | 3.31 |
| Aldh6a1 protein | Q8K0L1 | Q8K0L1 | 1.21 ± 0.7 | | | 4.46 ± 0.37 | **0.006** | 3.7 |
| 40S ribosomal protein SA < | P14206 | RSSA | 0.95 ± 0.6 | | | 3.56 ± 0.2 | **0.006** | 3.75 |
| Putative uncharacterized protein | Q3U9J9 | Q3U9J9 | 2.06 ± 0.24 | | | 7.75 ± 1.18 | **0.003** | 3.75 |
| Putative uncharacterized protein | Q3TKF8 | Q3TKF8 | 5.1 ± 1.94 | | | 20.81 ± 4.45 | **0.018** | 4.08 |
| Interleukin-17B | Q9QXT6 | IL17B | 3.51 ± 1.28 | | | 14.34 ± 2.03 | **0.004** | 4.09 |
| Treslin | Q8BQ33 | TICRR | 1.2 ± 0.7 | | | 5 ± 0.63 | **0.007** | 4.16 |
| **Table S1.** *Cont* | | | | | | | | |
| **Description** | **Protein-ID** | **Abbrev.** | **Vehicle** | | | **LP10** | ***p* value** | **Fold** |
| UDP-glucose 6-dehydrogenase | Q3UIZ1 | Q3UIZ1 | 0.81 ± 0.28 | | | 3.42 ± 0.28 | **0.001** | 4.25 |
| Gamma actin-like protein | Q9QZ83 | Q9QZ83 | 5.13 ± 2.57 | | | 22.15 ± 1.91 | **0.002** | 4.32 |
| Putative uncharacterized protein | Q3TUD6 | Q3TUD6 | 1.97 ± 1.16 | | | 9.04 ± 1.27 | **0.006** | 4.59 |
| Uncharacterized protein | F6YVP7 | F6YVP7 | 0.67 ± 0.39 | | | 3.11 ± 0.27 | **0.002** | 4.62 |
| UDP-glucuronosyltransferase 2A3 | Q8BWQ1 | UD2A3 | 1.23 ± 0.52 | | | 6.53 ± 0.96 | **0.003** | 5.31 |
| Peroxiredoxin-4 | O08807 | PRDX4 | 0.21 ± 0.21 | | | 1.11 ± 0.07 | **0.006** | 5.34 |
| Hypoxia up-regulated protein 1 | Q9JKR6 | HYOU1 | 3.26 ± 1.81 | | | 18.64 ± 1.14 | **0.000** | 5.71 |
| UDP-glucose 6-dehydrogenase | Q3TJE8 | Q3TJE8 | 0.81 ± 0.28 | | | 4.63 ± 1.11 | **0.016** | 5.75 |
| Zinc finger protein 76 | Q8BMU0 | ZNF76 | 1.75 ± 0.75 | | | 10.19 ± 1.87 | **0.006** | 5.83 |
| Putative uncharacterized protein | Q3UHS6 | Q3UHS6 | 0.42 ± 0.42 | | | 2.5 ± 0.61 | **0.030** | 6.02 |
| Talin-1 | P26039 | TLN1 | 0.42 ± 0.42 | | | 2.5 ± 0.61 | **0.030** | 6.02 |
| Very long-chain acyl-CoA synthetase | O35488 | S27A2 | 1.39 ± 1.39 | | | 9.23 ± 1.33 | **0.006** | 6.65 |
| Protein transport protein Sec23A | Q01405 | SC23A | 0.21 ± 0.21 | | | 1.42 ± 0.37 | **0.030** | 6.83 |
| Protein transport protein Sec23A | E9Q1S3 | E9Q1S3 | 0.21 ± 0.21 | | | 1.42 ± 0.37 | **0.030** | 6.83 |
| Putative uncharacterized protein | Q8C1E4 | Q8C1E4 | 0.21 ± 0.21 | | | 1.42 ± 0.37 | **0.030** | 6.83 |
| Fibrinogen gamma chain | Q8VCM7 | FIBG | 0.28 ± 0.28 | | | 2.06 ± 0.67 | **0.049** | 7.43 |
| Fibrinogen gamma chain | Q3UER8 | Q3UER8 | 0.28 ± 0.28 | | | 2.06 ± 0.67 | **0.049** | 7.43 |
| Protein disulfide-isomerase | Q3TIM0 | Q3TIM0 | 3.05 ± 1.07 | | | 24.21 ± 3.03 | **0.001** | 7.94 |
| Calnexin | P35564 | CALX | 1.26 ± 0.85 | | | 10.4 ± 1.3 | **0.001** | 8.22 |
| Putative uncharacterized protein | Q3TXE5 | Q3TXE5 | 1.26 ± 0.85 | | | 10.4 ± 1.3 | **0.001** | 8.22 |
| Corticosteroid 11-beta-dehydrogenase isozyme 1 | P50172 | DHI1 | 0.24 ± 0.24 | | | 2.14 ± 0.48 | **0.012** | 8.78 |
| Corticosteroid 11-beta-dehydrogenase isozyme 1 | Q3TJI8 | Q3TJI8 | 0.24 ± 0.24 | | | 2.14 ± 0.48 | **0.012** | 8.78 |
| Ribosomal protein L21 | Q4VA28 | Q4VA28 | 0.32 ± 0.33 | | | 3.4 ± 0.43 | **0.001** | 10.46 |
| 2-amino-3-ketobutyrate coenzyme A ligase, mitochondrial | E9PWY6 | E9PWY6 | 0 ± 0 | | | 1.17 ± 0.47 | **0.045** | 100 |
| 2-oxoglutarate dehydrogenase, mitochondrial | Q60597 | ODO1 | 0 ± 0 | | | 1.59 ± 0.63 | **0.046** | 100 |
| 60S ribosomal protein L27a | P14115 | RL27A | 0 ± 0 | | | 1.46 ± 0.5 | **0.027** | 100 |
| Acyl-coenzyme A oxidase | Q3UNF3 | Q3UNF3 | 0 ± 0 | | | 1.17 ± 0.42 | **0.031** | 100 |
| **Table S1.** *Cont* | | | | | | | | |
| **Description** | **Protein-ID** | **Abbrev.** | **Vehicle** | | | **LP10** | ***p* value** | **Fold** |
| Alpha glucosidase 2 alpha neutral subunit | A1A4T2 | A1A4T2 | 0 ± 0 | | | 2.83 ± 0.51 | **0.001** | 100 |
| Annexin | Q3V2Z4 | Q3V2Z4 | 0 ± 0 | | | 0.96 ± 0.37 | **0.040** | 100 |
| Annexin | Q8CEX0 | Q8CEX0 | 0 ± 0 | | | 0.96 ± 0.37 | **0.040** | 100 |
| Annexin A5 | P48036 | ANXA5 | 0 ± 0 | | | 0.75 ± 0.26 | **0.028** | 100 |
| Cytochrome c oxidase subunit 2 | A0A023J5Y1 | A0A023J5Y1 | 0 ± 0 | | | 0.96 ± 0.36 | **0.039** | 100 |
| Dipeptidyl peptidase 3 | Q99KK7 | DPP3 | 0 ± 0 | | | 0.81 ± 0.28 | **0.028** | 100 |
| Dipeptidylpeptidase 3 | Q6NXZ0 | Q6NXZ0 | 0 ± 0 | | | 0.81 ± 0.28 | **0.028** | 100 |
| Dipeptidylpeptidase 3, isoform CRA_c | Q8BU29 | Q8BU29 | 0 ± 0 | | | 0.81 ± 0.28 | **0.028** | 100 |
| Endoplasmic reticulum resident protein 44 | Q9D1Q6 | ERP44 | 0 ± 0 | | | 1.77 ± 0.3 | **0.001** | 100 |
| ES1 protein homolog, mitochondrial | Q9D172 | ES1 | 0 ± 0 | | | 0.89 ± 0.32 | **0.030** | 100 |
| Eukaryotic translation initiation factor 3 subunit B | Q8JZQ9 | EIF3B | 0 ± 0 | | | 0.95 ± 0.34 | **0.032** | 100 |
| Eukaryotic translation initiation factor 3 subunit B | Q8CIJ3 | Q8CIJ3 | 0 ± 0 | | | 0.95 ± 0.34 | **0.032** | 100 |
| Fatty-acid amide hydrolase 1 | O08914 | FAAH1 | 0 ± 0 | | | 1.49 ± 0.23 | **0.001** | 100 |
| Glucosidase 2 subunit beta | O08795 | GLU2B | 0 ± 0 | | | 1.19 ± 0.48 | **0.048** | 100 |
| Major vault protein | Q9EQK5 | MVP | 0 ± 0 | | | 1.62 ± 0.6 | **0.036** | 100 |
| Major vault protein | E9Q3X0 | E9Q3X0 | 0 ± 0 | | | 1.62 ± 0.6 | **0.036** | 100 |
| Myb protein | Q61926 | Q61926 | 0 ± 0 | | | 0.95 ± 0.32 | **0.026** | 100 |
| Myb proto-oncogene mRNA for c-myb protein | Q61421 | Q61421 | 0 ± 0 | | | 0.95 ± 0.32 | **0.026** | 100 |
| Neutral alpha-glucosidase AB | Q8BHN3 | GANAB | 0 ± 0 | | | 2.83 ± 0.51 | **0.001** | 100 |
| Peroxisomal acyl-coenzyme A oxidase 2 | Q9QXD1 | ACOX2 | 0 ± 0 | | | 1.17 ± 0.42 | **0.031** | 100 |
| Phosphomannomutase | Q9DCE3 | Q9DCE3 | 0 ± 0 | | | 1.04 ± 0.37 | **0.029** | 100 |
| Phosphomannomutase 2 | Q9Z2M7 | PMM2 | 0 ± 0 | | | 1.04 ± 0.37 | **0.029** | 100 |
| Proline synthetase co-transcribed | Q6P8V7 | Q6P8V7 | 0 ± 0 | | | 1.13 ± 0.44 | **0.043** | 100 |
| Putative uncharacterized protein | Q3THP1 | Q3THP1 | 0 ± 0 | | | 0.81 ± 0.28 | **0.028** | 100 |
| Putative uncharacterized protein | Q3THX5 | Q3THX5 | 0 ± 0 | | | 1.62 ± 0.6 | **0.036** | 100 |
| Putative uncharacterized protein | Q3TIZ0 | Q3TIZ0 | 0 ± 0 | | | 4.19 ± 0.65 | **0.001** | 100 |
| Putative uncharacterized protein | Q3TWG2 | Q3TWG2 | 0 ± 0 | | | 1.19 ± 0.48 | **0.048** | 100 |
| **Table S1.** *Cont* | | | | | | | | |
| **Description** | **Protein-ID** | **Abbrev.** | **Vehicle** | | | **LP10** | ***p* value** | **Fold** |
| Putative uncharacterized protein | Q3U3D3 | Q3U3D3 | 0 ± 0 | | | 1.86 ± 0.71 | **0.040** | 100 |
| Putative uncharacterized protein | Q3UDD5 | Q3UDD5 | 0 ± 0 | | | 0.81 ± 0.28 | **0.028** | 100 |
| Putative uncharacterized protein | Q3UDF3 | Q3UDF3 | 0 ± 0 | | | 0.81 ± 0.28 | **0.028** | 100 |
| Putative uncharacterized protein | Q8BRM1 | Q8BRM1 | 0 ± 0 | | | 1.49 ± 0.23 | **0.001** | 100 |
| Putative uncharacterized protein | Q8BU17 | Q8BU17 | 0 ± 0 | | | 0.95 ± 0.32 | **0.026** | 100 |
| Putative uncharacterized protein | Q8C2S9 | Q8C2S9 | 0 ± 0 | | | 1.62 ± 0.6 | **0.036** | 100 |
| Putative uncharacterized protein | Q8C541 | Q8C541 | 0 ± 0 | | | 1.49 ± 0.23 | **0.001** | 100 |
| Putative uncharacterized protein | Q9D9Y2 | Q9D9Y2 | 0 ± 0 | | | 1.11 ± 0.12 | **0.000** | 100 |
| Ribosome-binding protein 1 | Q99PL5 | RRBP1 | 0 ± 0 | | | 5.17 ± 1.74 | **0.025** | 100 |
| Ribosome-binding protein 1 | A2AVJ7 | A2AVJ7 | 0 ± 0 | | | 5.17 ± 1.74 | **0.025** | 100 |
| Transcriptional activator Myb | P06876 | MYB | 0 ± 0 | | | 0.95 ± 0.32 | **0.026** | 100 |
| Transcriptional activator Myb | A0A087WPA7 | A0A087WPA7 | 0 ± 0 | | | 0.95 ± 0.32 | **0.026** | 100 |
| Tubulin alpha-1A chain | P68369 | TBA1A | 0 ± 0 | | | 2.75 ± 0.4 | **0.001** | 100 |
| Tubulin alpha-1B chain | P05213 | TBA1B | 0 ± 0 | | | 4.62 ± 0.7 | **0.001** | 100 |
| Tubulin alpha-1C chain | P68373 | TBA1C | 0 ± 0 | | | 4.19 ± 0.65 | **0.001** | 100 |
| Tubulin alpha-3 chain | P05214 | TBA3 | 0 ± 0 | | | 1.11 ± 0.12 | **0.000** | 100 |
| Tubulin alpha-8 chain | Q9JJZ2 | TBA8 | 0 ± 0 | | | 1.79 ± 0.24 | **0.000** | 100 |
| Tumor-specific myb protein | Q61928 | Q61928 | 0 ± 0 | | | 0.95 ± 0.32 | **0.026** | 100 |
| Valine--tRNA ligase | Q9Z1Q9 | SYVC | 0 ± 0 | | | 1.86 ± 0.71 | **0.040** | 100 |
| Valyl-tRNA synthetase | Q7TPT7 | Q7TPT7 | 0 ± 0 | | | 1.86 ± 0.71 | **0.040** | 100 |
| Very long-chain-specific acyl-CoA dehydrogenase, mitochondrial | B1AR28 | B1AR28 | 0 ± 0 | | | 4.09 ± 1.12 | **0.010** | 100 |

^1^ Table S1 is a list of uniquely detected, significantly increased, non-detected, and significantly decreased liver proteome at LP10-treated, as ranked by *p*-value. *p* values were analyzed by paired Student’s *t*-test. ^2^ Data were expressed by Mean ± SEM from 4 independent samples. ^3^ Protein description, database name, and ID relate to the Swiss-Prot database (57.1) entry returned using the MASCOT search engine. A MOWSE score greater than 51 denotes a confident (*p* < 0.05) protein identification. Vehicle and LP10 expression ratios were the Mean ± SEM of peptides quantified in each sample expressed relative to the pooled internal standard. Fold difference relative to vehicle values was reported and *p-*values were determined from log-transformed data using Student’s independent *t*-tests. The complete list of proteins identified by iTRAQ and the Mean ± SEM number of MS/MS ions analyzed and the Mean ± SEM peptides that met the inclusion criteria for iTRAQ analysis (including amino acid sequences identified) were reported.
